# Electromyographic Assessment of Anterior Cruciate Ligament Injury Risk in Male Tennis Players: Which Role for Visual Input? A Proof-of-Concept Study

**DOI:** 10.3390/diagnostics11060997

**Published:** 2021-05-30

**Authors:** Alessandro de Sire, Nicola Marotta, Andrea Demeco, Lucrezia Moggio, Pasquale Paola, Marcello Marotta, Teresa Iona, Marco Invernizzi, Massimiliano Leigheb, Antonio Ammendolia

**Affiliations:** 1 Department of Medical and Surgical Sciences, University of Catanzaro “Magna Graecia”, 88100 Catanzaro, Italy; andreademeco@hotmail.it (A.D.); lucrezia.moggio@gmail.com (L.M.); ammendolia@unicz.it (A.A.); 2 Faculty of Applied Health Sciences, Brock University, St. Catharines, ON L2S3A1, Canada; pp19zy@brocku.ca; 3 Department of Animal Sciences, Food and Nutrition, Università Cattolica del Sacro Cuore, 29122 Piacenza, Italy; marcello.marotta@unicatt.it; 4 Department of Experimental and Clinical Medicine, University of Catanzaro “Magna Graecia”, 88100 Catanzaro, Italy; iona@unicz.it; 5 Physical and Rehabilitative Medicine, Department of Health Sciences, University of Eastern Piedmont, 28100 Novara, Italy; marco.invernizzi@med.uniupo.it; 6 Translational Medicine, Dipartimento Attività Integrate Ricerca e Innovazione (DAIRI), Azienda Ospedaliera Nazionale SS. Antonio e Biagio e Cesare Arrigo Alessandria, 15121 Alessandria, Italy; 7 Orthopaedics and Traumatology Unit, “Maggiore della Carità” Hospital, Department of Health Sciences, University of Eastern Piedmont, 28100 Novara, Italy; massimiliano.leigheb@med.uniupo.it

**Keywords:** knee injury, sports injury, tennis court, visual motor coordination, visual perception, rehabilitation

## Abstract

Anterior cruciate ligament (ACL) injury incidence is often underestimated in tennis players, who are considered as subjects conventionally less prone to knee injuries. However, evaluation of the preactivation of knee stabilizer muscles by surface electromyography (sEMG) showed to be a predictive value in the assessment of the risk of ACL injury. Therefore, this proof-of-concept study aimed at evaluating the role of visual input on the thigh muscle preactivation through sEMG to reduce ACL injury risk in tennis players. We recruited male, adult, semiprofessional tennis players from July to August 2020. They were asked to drop with the dominant lower limb from a step, to evaluate—based on dynamic valgus stress—the preactivation time of the rectus femoris (RF), vastus medialis, biceps femoris, and medial hamstrings (MH), through sEMG. To highlight the influence of visual inputs, the athletes performed the test blindfolded and not blindfolded on both clay and grass surfaces. We included 20 semiprofessional male players, with a mean age 20.3 ± 4.8 years; results showed significant early muscle activation when the subject lacked visual input, but also when faced with a less-safe surface such as clay over grass. Considering the posteromedial–anterolateral relationship (MH/RF ratio), tennis players showed a significant higher MH/RF ratio if blindfolded (22.0 vs. 17.0% not blindfolded; *p* < 0.01) and percentage of falling on clay (17.0% vs. 14.0% in grass; *p* < 0.01). This proof-of-principle study suggests that in case of absence of visual input or falling on a surface considered unsafe (clay), neuro-activation would tend to protect the anterior stress of the knee. Thus, the sEMG might play a crucial role in planning adequate athletic preparation for semiprofessional male athletes in terms of reduction of ACL injury risk.

## 1. Introduction

Tennis is commonly considered a safe and low-risk sport; however, acute injuries tend to occur in the lower extremities, while chronic overuse injuries often affect the upper extremities and trunk [1]. Ankle sprain is the most common acute injury [1], but racquet sports might also involve sharp, side-to-side movements, leading to significant valgus and rotatory stresses on the knee [2]. Indeed, the annual incidence of anterior cruciate ligament (ACL) injury in amateurs is underestimated compared to professionals, even in a sport conventionally less prone to knee injuries [3]. Furthermore, it has been shown that ACL injury may be related to higher risk of knee reinjury [4], with a consequently long-term disability and higher risk of early osteoarthritis [5].

To date, there is an existence of neuromuscular asymmetries in individuals with characteristic knee instability [6]. In this context, preactivation might reduce the injury probability, and the lower extremity musculature may be 40–80% activated when the foot touches the ground [7]. In this scenario, electromyography (EMG) neuromuscular evaluation provides insight into how the neuromuscular system behaves. Indeed, Medina et al. [8] recently assessed muscular preactivation of selected lower limb muscles in response to a drop landing, underlying the need to improve hamstring training methods to obtain better neuromuscular control, thus reducing the ACL injury risk. Specifically, it is possible to determine the timing of muscle activation, showing when a muscle turns “on” [9]. As neuromuscular agonists of ACL, the medial thigh muscles play a crucial role in injury risk reduction. Indeed, the preactivation of medial quadriceps and hamstrings appears to limit the risk of excessive dynamic valgus and external rotation of the knee [10,11]. Besides, player performance is affected by different surfaces [12], especially in sports such as tennis with grass and clay courts [13]. Serpell et al. suggested that medial hamstring–quadriceps coactivation may limit ACL elongation; although, if lateral activation exceeds medial, ACL elongation could be performed [14]. In this scenario, reduced surface EMG (sEMG) preactivation of medial hamstrings compared to EMG preactivation of lateral quadriceps during an ACL stress manoeuvre increases the anterior share stress and the risk of ACL injury [15]. On the assumption that in the brain–body–environment resonance, there were not only “sensory” notions of inputs and messages coming from an electromechanical system, but a nervous system that operates in circular circuits and in which information is never transmitted but extracted from the collection of invariants over time [16]. Thus, instead of reflexes or stimulus-response effects, these continuous feed circuits, through transactions of information with their environments, could resonate simultaneously in different structures of the nervous system, as well as resonate in the neuromuscular system, and even more widely through the environment–organism coupling [17]. The role of inertial movement units (IMU) and sEMG might be crucial in the assessment of muscle activity in adult subjects, considering that the dynamics of the brain–body–environment resonant model could facilitate continuous interactions in the performance environment [17,18]. In this context, it should also take into consideration that athletes might have psychological implications due to high-intensity training [19,20].

To date, the quadriceps–hamstring muscles’ coactivation supporting training has been described in literature to prevent ACL injuries [14,21], but there is a lack of studies assessing the influence that visual input and different surfaces could have during a stressful knee movement [14]. Therefore, the present proof-of-concept study aims to evaluate the role of visual input on thigh muscle preactivation through sEMG to reduce ACL injury risk in male tennis players.

## 2. Materials and Methods

### 2.1. Participants

In this proof-of-concept study, we included semiprofessional tennis players recruited from a Southern Italy Tennis Club, during a preseason phase from July to August 2020, who participated in the study voluntarily. Inclusion criteria were as follows: (a) male aged between 18 and 30 years; (b) participation at local agonistic competitions in the last 5 years; (c) experience lasting at least 5 years; d) SARS-CoV-2 negative swab test. Exclusion criteria were as follows: (a) traumatic knee injuries that required surgical intervention; (b) any acute injury to the back or lower extremity for at least two weeks prior to testing; (c) history of participation in any type of ACL injury prevention program.

Before the examination, all tennis players read and signed a written informed consent statement. The study was compliant with the ethical guidelines of the responsible governmental agency and was approved by the local Institutional Review Board. All researchers involved were instructed to protect the participants’ privacy, and the procedures were performed according to the Declaration of Helsinki.

### 2.2. Electromyography

**Figure 1:**
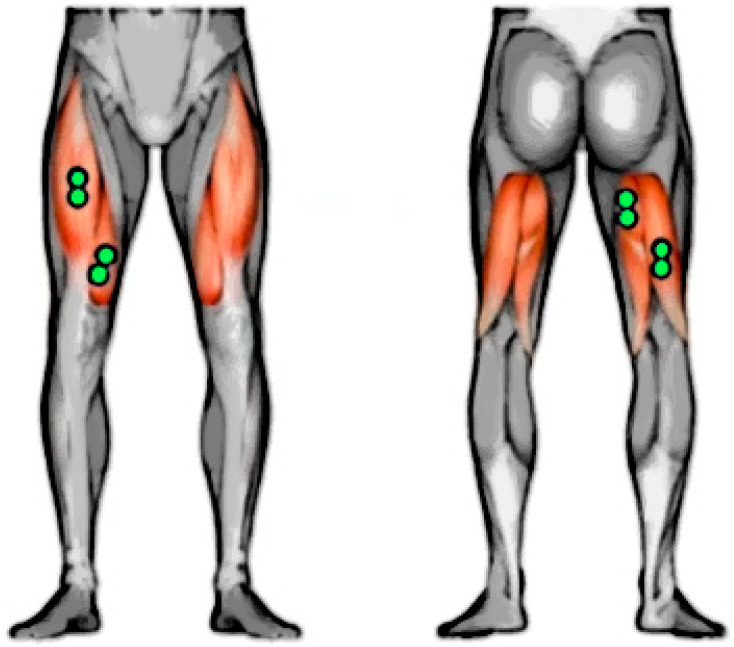
The bipolar surface electrodes were placed on Rectus Femoris (RF), Vastus Medialis (VM), Medial Hamstring (MH), and Biceps Femoris (BF) muscles (from left to right of the image).

The sEMG was performed with a wireless EMG device (FREE1000 BTS Bioengineering, Milano, Italy) using bipolar surface electrodes (diameter, 0.8 cm; interelectrode distance, 2 cm; pregelled disposable, surface Ag/AgCl Ambu Neuroline 720 electrodes (Ambu, Neuroline, Ballerup, Denmark) [22,23,24,25]. The sEMG electrodes were placed by an experienced physician on the Rectus Femoris (RF), Vastus Medialis (VM), Biceps Femoris (BF), and Medial Hamstring (MH) muscles. Before performing the sEMG, the surface of the skin was shaved, gently abraded, and cleaned with alcohol to reduce the impedance of the skin [22]. The surface disc electrodes were positioned according to the sEMG recommendations for Not-Invasive Assessment of Muscles (SENIAM) [25] (see Figure 1 for further details). 

The raw sEMG data were recorded with a sample frequency of 1000 Hz. All recorded signals were band-pass filtered using a high- and low-pass Hamming filter with cut-off frequencies of 10 and 500 Hz, respectively, and an additional 50 Hz 80 dB/decade notch filter. The signals were rectified, and a low-pass 5 Hz Hamming filter was used to calculate the linear envelope to represent the amplitude values of the EMG signals. Kinematic data were captured using an IMU (G-sensor, BTS Bioengineering Spa, Garbagnate M.se—Milano, ITA). The wireless IMU was attached to the trunk with an elastic belt; it was used to determine initial ground contact, and the onset of muscle activity for each trial was referenced to that point of initial ground contact. The sEMG and IMU signals were processed by the software sEMG Analyzer (BTS Bioengineering Spa, Garbagnate M.se—Milano, ITA), applying the “drop landing test” protocol. 

### 2.3. Drop Landing Test

All participants performed a “drop landing test” according to Medina et al. [8], who first described this protocol in young adults. To perform the test correctly, all subjects first performed a warm-up program with an experienced instructor [8,11,26]. The sEMG and IMU probes were positioned as described in the previous paragraph, and each participant was positioned on the edge of a 32-cm platform with the test leg suspended from the step. To perform the test, at an agreed signal, each tennis player shifted their weight forward and dropped vertically, attempting to land in a balanced position on the test leg without jumping or bending [8,11]. At the beginning of each “drop landing test”, a quiet period during which there was no discernible muscle activity was recorded and used as a baseline. All athletes performed three trials, which were visually monitored to verify that the technique was being performed correctly. Failed trials were discarded and repeated. For each subject, the two trials with the least variation were averaged for data analysis. 

In this proof-of-principle study, the “drop landing test” was planned to be performed by the semiprofessional tennis players to analyze the influence of visual input on the study participants in different environmental conditions.

Firstly, we assessed the impact of the presence of visual input, where each participant performed the test blindfolded and nonblindfolded on a clay surface. 

**Figure 2:**
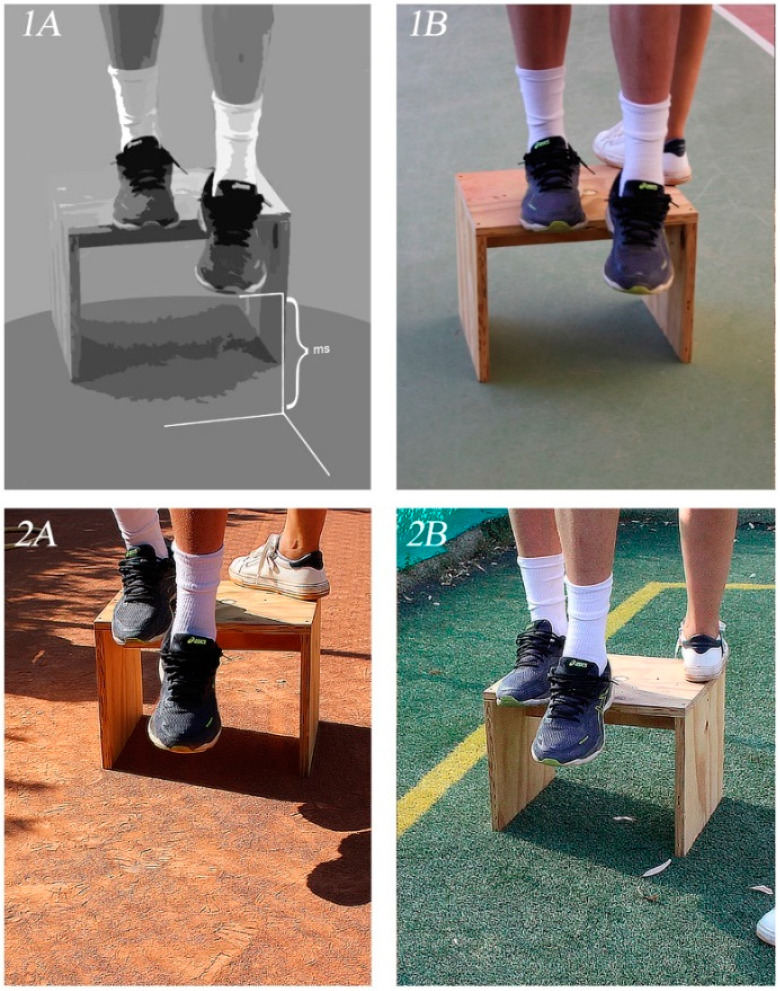
A patient landed blindfolded (**1A**) and not blindfolded (**1B**), then landed on clay (**2A**) and grass (**2B**).

Secondly, the athletes performed the “drop landing test”, influenced by the visual input of different tennis court surfaces: clay and grass (see Figure 2 for further details).

### 2.4. Outcome Measures

The same physician experienced in sEMG interpretation calculated the preactivation times of RF, VM, BF, and MH muscles as an outcome measure. In more detail, it was the time interval between the moment of activation determined by the sEMG envelope of the muscle and the time of contact with the ground. Furthermore, the preactivation ratio between the medial hamstring muscles and the rectus femoris (MH/RF) was assessed [10]. All outcome measures were evaluated based on the different visual input (blindfolded vs. nonblindfolded; clay tennis court vs. grass tennis court) to demonstrate any muscle imbalances and potential risk of injury.

### 2.5. Statistical Analysis

Data were analyzed using R (version 3.5.1; R Foundation, Vienna, Austria). First, we tested the data for normality using the Shapiro–Wilk normality test. Due to the non-normal distribution of the preactivation muscle time, the Wilcoxon signed-rank test was performed. On the other hand, there was a normal distribution of the MH/RF ratios, thus allowing us to perform paired samples t-tests. The continuous values were presented as means ± standard deviations. An α level of 5% was accepted as statistically significant. Furthermore, reliability was calculated with intraclass correlation coefficients (ICC), using average data (mean of three tests per session) with a 95% confidence interval (CI). The interpretation of ICC values showed good reproducibility for ICC values ranging from 0.60 to 0.79, and very good reproducibility for ICC values from 0.80 to 1.

## 3. Results

Of the 22 semiprofessional male players recruited, two of them did not meet the eligibility criteria, due to injuries to the lower limbs that occurred in the previous two weeks. Thus, 20 subjects, with a mean age of 20.3 ± 4.8 years and mean body mass index of 22.9 ± 1.5 kg/m^2^ were included as study participants. Results show high reproducibility: test–retest of 0.90 (0.87–0.92) and inter-rater of 0.87 CI (0.83–0.91) for all the measurements analyzed. Higher preactivity of the muscles examined without visual input was observed with subjects blindfolded rather than nonblindfolded (*p* <0.01), and when the player falls on clay rather than on grass (*p* < 0.01) (Table 1). 

An early muscle activation was noted when the subject lacked visual input, but also when faced with a surface considered unsafe such as clay or grass. Furthermore, the activation of MH with RF was compared to understand how the risk of generating anterior stress on the knee changed as a posteromedial–anterolateral relationship (MH/RF ratio). 

**Figure 3:**
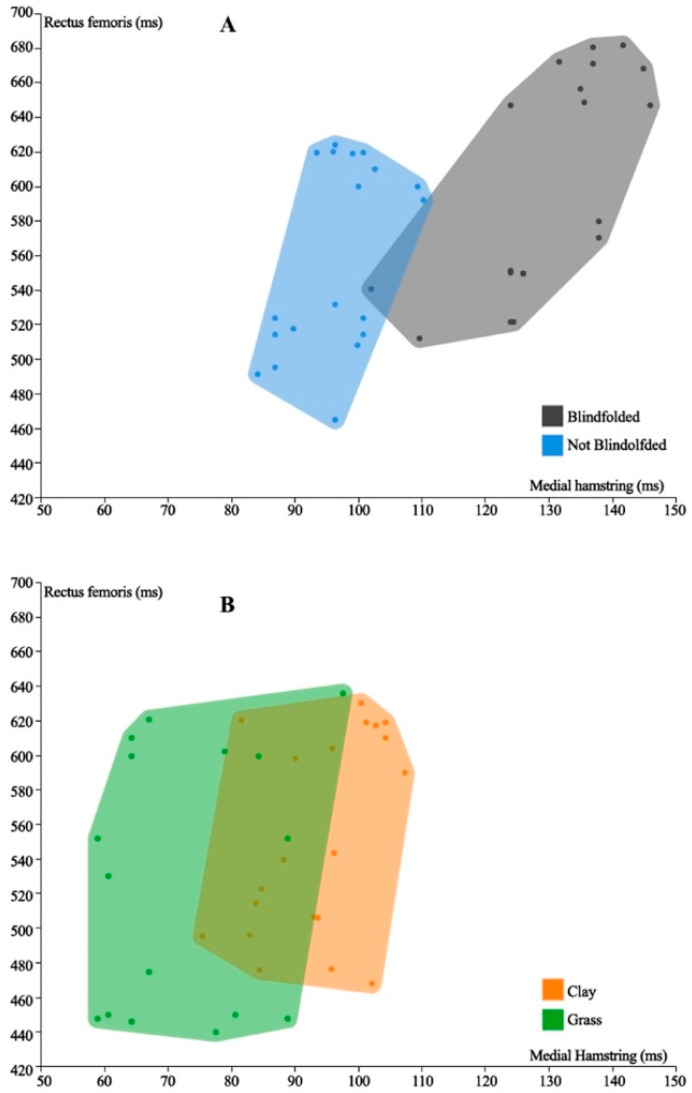
Posteromedial–anterolateral ratio of muscle preactivation with or without visual input (**A**) and landing on clay or grass surfaces (**B**).

The ratio of preactivation of MH to RF is 22.0% in blindfolded subjects versus 17.0% when not blindfolded (*p* < 0.01), showing higher preactivation in medial compared to lateral quadriceps when the subjects were not blindfolded (Figure 3A). Similarly, a significant difference in the MH/RF ratio was demonstrated when the individual fell on clay versus grass (17% vs. 14%; *p* < 0.01) (Figure 3B). When the blindfolded athlete faces a surface considered unsafe, the muscles tend to protect from anterior stress by activating the MH in relation to the RF.

## 4. Discussion

This study aimed to evaluate the influence of visual conditioning on the neuromuscular activation of knee stabilizers in tennis players. When blindfolded, tennis players recruit muscles earlier than when not blindfolded. On the clay court, all subjects recruited muscles much earlier than on grass ground. Hence, the lack of vision of the landing surface and the perception of an unsafe court appears to influence early muscle recruitment to protect players from potential injuries. 

Individuals seem to develop—under a hood of ecological neurodynamic inputs—a range of skills honed through continuous interactions with the environment and live under the constraints of both [18]. A neuroscience model integrated with cognition, perception, and action in the context of sports reveals a continuous brain–body–environment adaptive modulation in the psychological, physical, and emotional capacities of performance [27,28]. In addition to several risk factors, neuromuscular control appears to play an important role in knee integrity [29]. Heinrich stated that insufficient or slow hamstring reaction might result in inadequate knee stabilization during sporting tasks involving large external loads and increased risk of ACL injury [30]. Athletes with ACL reconstruction have shown altered neuromuscular response, which could be an arthrogenic muscle response, demonstrating additional adaptation to disrupted activities compared to controls, potentially altered proprioceptive input [31]. Therefore, it becomes crucial to evaluate the activation patterns to prevent an ACL injury and not only examine it when the injury occurs [32]. Late activation of MH [10] and VM [11] during side-cutting predisposes the potential risk of ACL injury. Analyzing the raw activation times allows us to have an overview of the knee stabilizers [8]. In more detail, the ratio of MH/RF activation could guarantee an optimal index to evaluate when the posterior agonist is activated compared to the anterior antagonist [10,33]. More specifically, college tennis injury rates were similar overall between genders and were higher during match play than during training [34]. Additionally, racquet sports require sharp side-to-side shifts and impose significant valgus rotational forces on the knee [35,36], and the playing surface significantly affects lower limb loading during tennis activities [37].

For tennis players, the clay court is a slower surface than grass [38], due to its higher coefficient of friction and restitution [39]. The intrinsic characteristics of a surface appear to be related to the different risk of injury, because a decrease in turf tournaments seems to lead to an increase in the number of player injuries [13,37]. Barnett et al. recently reported fewer unfinished tennis matches on grass courts than clay courts in professional Grand Slam tournaments [39].

Several authors have reported a higher percentage of dropouts during matches on clay courts than on grass surfaces, as well as a potential different risk of injury based on tennis-specific courses [37,40]. Landing on a surface considered less safe results in feed-forward conditioning for muscle recruitment to stabilize the knee before a stressful movement. On the other hand, approaching a safer tennis court would seem to lower this protection threshold, with consequent worsening of the neuromuscular defense of the knee in a situation considered “safer”.

Paradoxically, in the present study, the visual inputs analyzed seem to affirm that surface safety does not translate into safe training. Changing training surfaces has proved to be a viable strategy to improve a player’s rehabilitation, as it has been hypothesized that exercises on less-safe surfaces recruit more muscle fibers than those performed on grass [41].

This study presented some limitations: first, the small sample size with no power statistics, although this is a proof-of-concept study; second, muscle mechanics and electromechanical delay [42] can affect the test values, also considering that other muscles could affect the knee’s dynamic stability; third, the different training status of the athletes could not be excluded, although the study was conducted in preseason and the COVID-19 pandemic did not allow participants to train and compete properly; fourth, the study did not use three-dimensional analyses of dynamic valgus [26]; lastly, the preactivation time and the MH/RF ratio remain indicative risk factors for ACL injury [8].

## 5. Conclusions

Taken together, results of this proof-of-concept study suggest that a more protective neuromuscular physical approach could be useful in semiprofessional male tennis players playing on “less-safe” surfaces. In this context, the sEMG assessment might play a crucial role to plan and carry on adequate physical exercise and specific proprioceptive training aimed at ACL injuries. Further studies on larger samples of athletes are warranted to confirm our data and to plan a series of exercises for tennis players to avoid ACL injuries.

## Figures and Tables

**Table 1:** Differences in preactivation time (ms) of knee stabilizer muscles.

	Presence or Absence of Visual Input			Different Tennis Court Surfaces		
	Blindfolded	Not Blindfolded	*p* Value	Clay	Grass	*p* Value
RF (ms)	598.2 ± 62.9	552.5 ± 57.6	*p* < 0.01 *	552.4 ± 58.7	513.4 ± 74.3	*p* < 0.01 *
VM (ms)	204.9 ± 11.6	190.6 ± 12.2	*p* < 0.01 *	190.6 ± 12.2	137.3 ± 12.3	*p* < 0.01 *
BF (ms)	145.1 ± 9.1	127.9 ± 7.6	*p* < 0.01 *	125.9 ± 9.1	83.2 ± 12.3	*p* < 0.01 *
MH (ms)	129.9 ± 11.0	96.9 ± 7.1	*p* < 0.01 *	93.5 ± 9.1	72.4 ± 12.1	*p* < 0.01 *
MH/RF	0.22 ± 0.02	0.177 ± 0.02	*p* < 0.01 *	0.17 ± 0.02	0.14 ± 0.03	*p* < 0.01 *

All variables are expressed as means ± standard deviations. Statistical analysis performed to assess intragroup differences was Wilcoxon signed-rank test. Paired *t*-test was used for comparison of MH/RF ratio. Abbreviations: RF—rectus femoris; VF—vastus medialis; BF—biceps femoris; MH—medial hamstrings; * = Statistical significance.

## Data Availability

Dataset is available on request.

## References

[1] Fu M.C., Ellenbecker T.S., Renstrom P.A., Windler G.S., Dines D.M. (2018). Epidemiology of injuries in tennis players. Curr. Rev. Musculoskelet. Med..

[2] Prieske O., Muehlbauer T., Krueger T., Kibele A., Behm D., Granacher U. (2015). Sex-specific effects of surface instability on drop jump and landing biomechanics. Int. J. Sports Med..

[3] Moses B., Orchard J., Orchard J. (2012). Systematic review: Annual incidence of ACL injury and surgery in various populations. Res. Sports Med..

[4] Waldén M., Hägglund M., Magnusson H., Ekstrand J. (2011). Anterior cruciate ligament injury in elite football: A prospective three-cohort study. Knee Surg. Sport. Traumatol. Arthrosc..

[5] Iolascon G., Gimigliano F., Moretti A., de Sire A., Migliore A., Brandi M.L., Piscitelli P. (2017). Early osteoarthritis: How to define, diagnose, and manage. A systematic review. Eur. Geriatr. Med..

[6] Gardinier E.S., Manal K., Buchanan T.S., Snyder-Mackler L. (2012). Gait and neuromuscular asymmetries after acute anterior cruciate ligament rupture. Med. Sci. Sports Exerc..

[7] Smeets A., Malfait B., Dingenen B., Robinson M.A., Vanrenterghem J., Peers K., Nijs S., Vereecken S., Staes F., Verschueren S. (2019). Is knee neuromuscular activity related to anterior cruciate ligament injury risk? A pilot study. Knee.

[8] Medina J.M., Valovich McLeod T.C., Howell S.K., Kingma J.J. (2008). Timing of neuromuscular activation of the quadriceps and hamstrings prior to landing in high school male athletes, female athletes, and female non-athletes. J. Electromyogr. Kinesiol..

[9] Vigotsky A.D., Halperin I., Lehman G.J., Trajano G.S., Vieira T.M. (2018). Interpreting signal amplitudes in surface electromyography studies in sport and rehabilitation sciences. Front. Physiol..

[10] Zebis M.K., Andersen L.L., Bencke J., Kjær M., Aagaard P. (2009). Identification of athletes at future risk of anterior cruciate ligament ruptures by neuromuscular screening. Am. J. Sports Med..

[11] Marotta N., Demeco A., de Scorpio G., Indino A., Iona T., Ammendolia A. (2020). Late activation of the vastus medialis in determining the risk of anterior cruciate ligament injury in soccer players. J. Sports Rehabil..

[12] Youdas J.W., Hollman J.H., Hitchcock J.R., Hoyme G.J., Johnsen J.J. (2007). Comparison of hamstring and quadriceps femoris electromyographic activity between men and women during a single-limb squat on both a stable and labile surface. J. Strength Cond. Res..

[13] Girard O., Micallef J.P., Millet G.P. (2010). Effects of the playing surface on plantar pressures during the first serve in tennis. Int. J. Sports Physiol. Perform..

[14] Serpell B.G., Scarvell J.M., Pickering M.R., Ball N.B., Newman P., Perriman D., Warmenhoven J., Smith P.N. (2015). Medial and lateral hamstrings and quadriceps co-activation affects knee joint kinematics and ACL elongation: A pilot study. BMC Musculoskelet. Disord..

[15] de Sire A., Demeco A., Marotta N., Moggio L., Palumbo A., Iona T., Ammendolia A. (2021). Anterior Cruciate Ligament Injury Prevention Exercises: Could a Neuromuscular Warm-Up Improve Muscle Pre-Activation before a Soccer Game? A Proof-of-Principle Study on Professional Football Players. Appl. Sci..

[16] Gibson J.J. (1979). The Ecological Approach to Visual Perception: Classic Edition.

[17] Badcock P.B., Friston K.J., Ramstead M.J.D., Ploeger A., Hohwy J. (2019). The hierarchically mechanistic mind: An evolutionary systems theory of the human brain, cognition, and behavior. Cogn. Affect. Behav. Neurosci..

[18] Negrini F., de Sire A., Lazzarini S.G., Pennestrì F., Sorce S., Arienti C., Vitale J.A. (2021). Reliability of activity monitors for physical activity assessment in patients with musculoskeletal disorders: A systematic review. J. Back Musculoskelet. Rehabil..

[19] Piacentini M.F., Minganti C., Ferragina A., Ammendolia A., Capranica L., Cibelli G. (2015). Stress related changes during a half marathon in master endurance athletes. J. Sports Med. Phys. Fit..

[20] Segura-García C., Papaianni M.C., Caglioti F., Procopio L., Nisticò C.G., Bombardiere L., Ammendolia A., Rizza P., De Fazio P., Capranica L. (2012). Orthorexia nervosa: A frequent eating disordered behavior in athletes. Eat. Weight Disord..

[21] Sherman D.A., Glaviano N.R., Norte G.E. (2021). Hamstrings Neuromuscular Function After Anterior Cruciate Ligament Reconstruction: A Systematic Review and Meta-Analysis. Sports Med..

[22] Sacco I.C.N., Gomes A.A., Otuzi M.E., Pripas D., Onodera A.N. (2009). A method for better positioning bipolar electrodes for lower limb EMG recordings during dynamic contractions. J. Neurosci. Methods.

[23] Miljković N., Malešević N., Kojić V., Bijelić G., Keller T., Popović D.B. (2015). Recording and assessment of evoked potentials with electrode arrays. Med. Biol. Eng. Comput..

[24] Demeco A., Marotta N., Moggio L., Pino I., Marinaro C., Barletta M., Petraroli A., Palumbo A., Ammendolia A. (2021). Quantitative analysis of movements in facial nerve palsy with surface electromyography and kinematic analysis. J. Electromyogr. Kinesiol..

[25] Hermens H.J., Freriks B., Disselhorst-Klug C., Rau G. (2000). Development of recommendations for SEMG sensors and sensor placement procedures. J. Electromyogr. Kinesiol..

[26] Marotta N., Demeco A., Moggio L., Isabello L., Iona T., Ammendolia A. (2020). Correlation between dynamic knee valgus and quadriceps activation time in female athletes. J. Phys. Educ. Sport.

[27] Seifert L., Button C., Davids K. (2013). Key properties of expert movement systems in sport: An ecological dynamics perspective. Sports Med..

[28] Araújo D., Davids K., Renshaw I. (2020). Cognition, Emotion and Action in Sport. Handbook of Sport Psychology.

[29] Myer G.D., Ford K.R., Brent J.L., Hewett T.E. (2007). Differential neuromuscular training effects onACL injury risk factors in “high-risk” versus “low-risk” athletes. BMC Musculoskelet. Disord..

[30] Heinrich D., van den Bogert A.J., Csapo R., Nachbauer W. (2020). A model-based approach to predict neuromuscular control patterns that minimize ACL forces during jump landing. Comput. Methods Biomech. Biomed. Engin..

[31] Smeets A., Verschueren S., Staes F., Vandenneucker H., Claes S., Vanrenterghem J. (2021). Athletes with an ACL reconstruction show a different neuromuscular response to environmental challenges compared to uninjured athletes. Gait Posture.

[32] Badiola-Zabala A., Massó-Ortigosa N., Cabedo-Sanromà J., Rey-Abella F., Milà R., Oviedo G.R. (2020). Observational study with the objective of determining possible correlations between GRF and muscle activation at reception after a jump in an ACL injury. Apunt. Sports Med..

[33] Nedergaard N.J., Dalbø S., Petersen S.V., Zebis M.K., Bencke J. (2020). Biomechanical and neuromuscular comparison of single- and multi-planar jump tests and a side-cutting maneuver: Implications for ACL injury risk assessment. Knee.

[34] Minghelli B., Cadete J. (2019). Epidemiology of musculoskeletal injuries in tennis players: Risk factors. J. Sports Med. Phys. Fitness..

[35] Majewski M., Susanne H., Klaus S. (2006). Epidemiology of athletic knee injuries: A 10-year study. Knee.

[36] Martin C., Sorel A., Touzard P., Bideau B., Gaborit R., DeGroot H., Kulpa R. (2020). Influence of the forehand stance on knee biomechanics: Implications for potential injury risks in tennis players. J. Sports Sci..

[37] Girard O., Eicher F., Fourchet F., Micallef J.P., Millet G.P. (2007). Effects of the playing surface on plantar pressures and potential injuries in tennis. Br. J. Sports Med..

[38] Clarke J., Carré M., Damm L., Dixon S. (2012). The influence of surface characteristics on the tribological interactions at the shoe-surface interface in tennis. Procedia Eng..

[39] Barnett T., Pollard G. (2007). How the tennis court surface affects player performance and injuries. Med. Sci. Tennis.

[40] Breznik K., Batagelj V. (2012). Retired matches among male professional tennis players. J. Sports Sci. Med..

[41] Rafols Parellada L., Linde X., Solà J., Fort A., Brau J. (2020). Muscle activation during rehabilitation on artificial turf vs. sand after cruciate ligament surgery: A case series. Apunt. Sports Med..

[42] Jacunski M., Rafferty G.F. (2020). The effects of hypoxia and fatigue on skeletal muscle electromechanical delay. Exp. Physiol..

